# Five-Year Trends in SSRI Consumption: A Precision Medicine Approach to Comparative Analysis Between Serbia and European Countries

**DOI:** 10.3390/healthcare13101174

**Published:** 2025-05-18

**Authors:** Janko Samardžić, Filip Simović, Kristina Sekanić, Milica Branković

**Affiliations:** Institute of Pharmacology, Clinical Pharmacology and Toxicology, Faculty of Medicine, University of Belgrade, 11000 Belgrade, Serbia; medsimovicf@gmail.com (F.S.); sekanic.kristina@gmail.com (K.S.); milica.radosavljevic@med.bg.ac.rs (M.B.)

**Keywords:** depression, anxiety, antidepressants, SSRIs, pharmacoepidemiology

## Abstract

**Background/Objectives**: Mental diseases are one of the leading groups of health disorders worldwide, with depressive and anxiety disorders being the most prevalent. Depressive disorders can be treated with pharmacotherapy, psychotherapy, or a combination of both. In cases where these approaches prove ineffective, electroconvulsive therapy may be considered as an alternative. The drugs of choice for treating depressive disorders are selective serotonin reuptake inhibitors (SSRIs). In the Republic of Serbia, commonly prescribed SSRIs include fluoxetine, citalopram, paroxetine, sertraline, and escitalopram. **Methods**: Data on drug sales for human medicine from the Agency for Medicines and Medical Devices of Serbia (ALIMS) were used for the analysis of consumption in the period 2018–2022. Data on drug consumption in other European countries were obtained from the respective national registers. **Results**: From 2018 to 2021, sertraline was the best-selling drug in this group, but with a statistically significant decrease (R^2^ = 0.7948, *p* = 0.042), while escitalopram showed a statistically significant increase (*p* = 0.006) and became the best-selling drug in the SSRI group in 2022. Overall, SSRI group consumption fluctuated from 2018 to 2022, with the highest values in 2020. However, these variations were not statistically significant (*p* = 0.6223). Compared to Serbia, out of 12 European countries, 8 had higher and 4 had lower consumption in 2019 and 2020. A positive correlation was found between antidepressant consumption and GDP per capita. **Conclusions**: Sertraline was the most commonly prescribed SSRI drug in Serbia from 2018 to 2021. However, in 2022, escitalopram became the most commonly used drug in this group both in Serbia and worldwide, with a consistent increase in consumption.

## 1. Introduction

Mental illnesses are among the leading global health challenges [1,2], with depressive and anxiety disorders being the most prevalent [3]. They also represent a significant burden on the population, ranking second after cardiovascular diseases in terms of disability-adjusted life years (DALY) and years lived with disability (YLD) [4]. Depressive disorders are the most common within this group, and the true prevalence is likely underestimated due to undiagnosed cases. Projections indicate that by 2030, depression will become the leading cause of YLD worldwide [5]. In severe cases, depression can result in suicide, which is the fourth most common cause of death among individuals aged 15 to 29 years [6].

Depression is a multifactorial disorder, with genetic, hormonal, and biochemical factors playing significant roles in its development, along with risk factors such as childhood abuse and neglect, the presence of chronic illnesses, and living conditions [7]. Epidemiological studies indicate that approximately one-third of the global population experiences an anxiety disorder at some point in their lives, with significantly higher prevalence rates reported in North and South America, Western and Central Europe, and Australia, compared to other regions [8]. Anxiety disorders are associated with a significant degree of disability and often occur as comorbidities with other mental illnesses.

The treatment of depressive disorders typically involves pharmacotherapy, psychotherapy, or a combination of both. In cases where these approaches prove ineffective, electroconvulsive therapy (ECT) may be considered as an alternative. Antidepressants, a cornerstone of pharmacological treatment, are classified based on their structure and mechanism of action. These include tricyclic and tetracyclic antidepressants (TCAs), monoamine oxidase inhibitors (MAOIs), selective serotonin reuptake inhibitors (SSRIs), serotonin–norepinephrine reuptake inhibitors (SNRIs), norepinephrine reuptake inhibitors (NaRIs), norepinephrine–dopamine reuptake inhibitors (NDRIs), serotonin antagonist and reuptake inhibitors (SARIs), and noradrenergic and specific serotonergic receptor antagonists (NASSAs), as well as newer antidepressants, including agomelatine and vortioxetine.

SSRIs are considered a first-line pharmacological treatment for depressive disorders. In the Republic of Serbia, commonly prescribed SSRIs include fluoxetine, citalopram, paroxetine, sertraline, and escitalopram. Their therapeutic indications and pharmacokinetic profiles are presented in Table 1 and Table 2. The most significant adverse effects of SSRIs include activation syndrome (psychomotor agitation, restlessness, tension and irritability, insomnia, and panic attacks) [9] and discontinuation syndrome (paresthesia, vertigo, lethargy, headache, sweating, insomnia and nightmares, nausea, vomiting, diarrhea, and extrapyramidal symptoms) [10], as well as sexual dysfunction (reduced libido, delayed orgasm or anorgasmia, and delayed ejaculation) [11,12]. Additionally, the concurrent use of SSRIs and MAOIs is contraindicated due to the risk of serotonin syndrome, a potentially life-threatening condition characterized by restlessness, myoclonus, hyperreflexia, tremors, hyperthermia, hypertension, seizures, and death [13].

The aim of this study is to determine the trend of SSRI consumption in Serbia from 2018 to 2022, as well as to compare this trend with SSRI consumptions in other European countries.

## 2. Materials and Methods

For the analysis of SSRI consumption from 2018 to 2022, data on the sale of medicines for human use were obtained from the Medicines and Medical Devices Agency of Serbia (ALIMS). The ATC/DDD methodology, recommended by the World Health Organization (WHO), was applied. According to the Anatomical Therapeutic Chemical (ATC) classification, SSRIs belong to the main anatomical group of drugs affecting the nervous system, specifically within group N06-psychoanaleptics and subgroup N06A-antidepressants.

The consumption analysis focused on the most commonly used oral formulations (tablets and capsules) covering five antidepressants from the SSRI group: fluoxetine, citalopram, paroxetine, sertraline, and escitalopram. The consumption indicator used was the number of defined daily doses per 1000 inhabitants per day (DDD/TID).

The total population of the Republic of Serbia in the corresponding year was obtained from the data provided by the Statistical Office of the Republic of Serbia.

The values for drug consumption in European countries were obtained from the respective national registers of Italy (*Agenzia Italiana del Farmaco*), Iceland (*Embætti landlæknis*), Spain (*Agencia Española de Medicamentos y Productos Sanitarios*), Croatia (*Agencija za lijekove i medicinske proizvode*), Norway (*Folkehelseinstituttet*), the Netherlands (*Zorgintituut Nederland*), Estonia (*Ravimiamet*), Finland (*Fimea*), Latvia (*Zāļu valsts aģentūra*), Lithuania (*Valstybinė vaistų kontrolės tarnyba prie LR Sveikatos Apsaugos Ministerijos*), Slovenia (*Nacionalni inštitut za javno zdravje*), and Denmark (*Sundhedsdatastyrelsen*). European countries included in this study were selected based on the availability and accessibility of online databases providing reliable data on pharmaceutical consumption.

Data on GDP per capita were retrieved from the Eurostat database. For data analysis, Microsoft Excel (Microsoft Corp., Redmond, WA, USA) and EZR (Saitama Medical Center, Jichi Medical University, Saitama, Japan) were used. Excel was used for data cleaning and basic tabulation, while EZR was used for inferential statistics and regression analysis. Parametric and non-parametric tests, Spearman’s correlation test, and linear regression were used in the statistical analysis.

## 3. Results

From 2018 to 2021, sertraline was the best-selling SSRI drug in the Republic of Serbia. However, its consumption showed a statistically significant decline (R^2^ = 0.7948, *p* = 0.042). Over the same period, escitalopram showed a statistically significant increase (*p* = 0.006), becoming the best-selling SSRI in 2022. Citalopram, the least-prescribed drug in this group, also experienced a statistically significant decline (*p* = 0.007).

**Figure 1:**
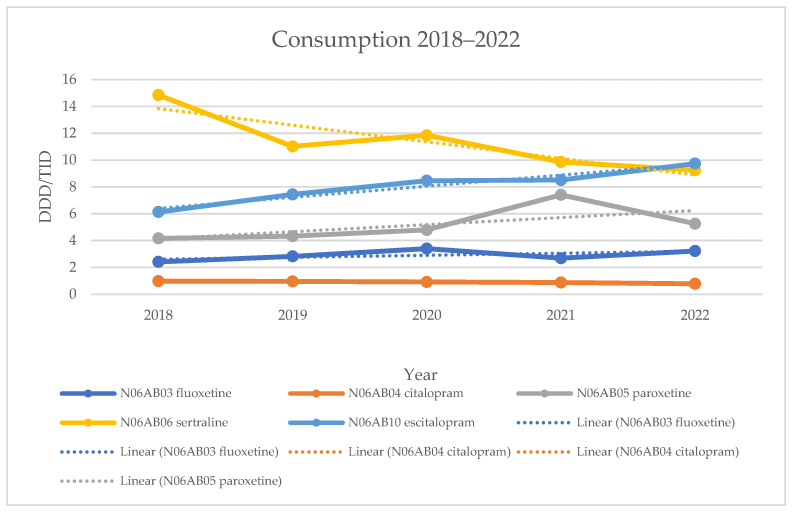
Consumption of antidepressants from the SSRI group in the period 2018–2022 in the Republic of Serbia.

All consumption data are presented in Table 3 and Table 4 and in Figure 1.

The overall consumption of SSRI drugs fluctuated between 2018 and 2022, reaching its highest values in 2020. However, these changes were not statistically significant (*p* = 0.6223).

**Figure 2:**
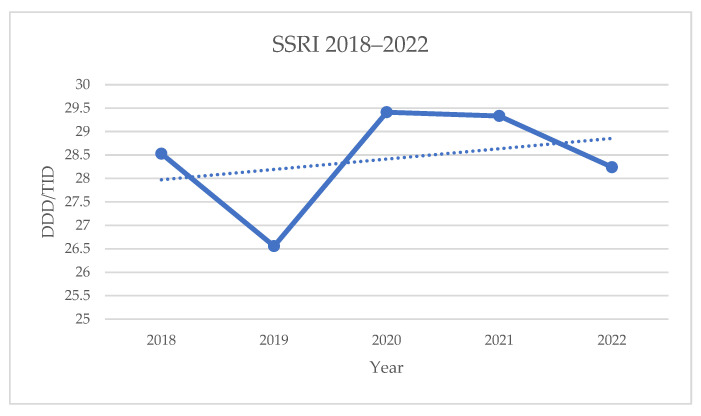
Annual consumption of SSRIs in the Republic of Serbia.

The annual SSRI consumption data are presented in Table 5 and Figure 2.

The obtained SSRI consumption values for the Republic of Serbia were compared to the consumption values in 12 European countries (Italy, Iceland, Spain, Croatia, Norway, the Netherlands, Estonia, Finland, Latvia, Lithuania, Slovenia, and Denmark) (Table 6).

**Figure 3:**
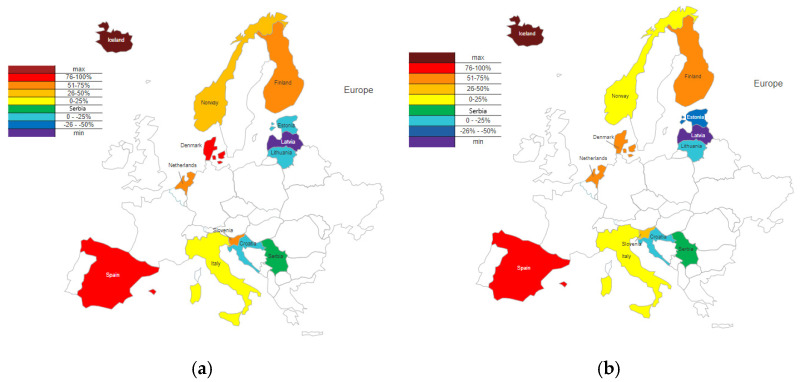
The difference in annual SSRI group consumption in the Republic of Serbia and 12 European countries in 2019 (**a**) and 2020 (**b**).

These data were compared with SSRI consumption in Serbia and expressed as percentages. The percentage differences were calculated and graphically presented for 2019, the year with the lowest consumption in Serbia, and 2020, the year with the highest consumption. Countries that had lower consumption than Serbia were divided into two groups: Group 1 (0 to −25%) and Group 2 (−25.1 to −50%), while countries that had higher consumption were divided into four groups: Group 1 (0 to 25%), Group 2 (25.1% to 50%), Group 3 (50.1% to 75%), and Group 4 (more than 75%). The distribution is presented in Figure 3. The difference in SSRI consumption across various European countries was highly statistically significant.

The individual consumptions of SSRI drugs in Serbia, Denmark, Spain, and Latvia in 2019 and 2020 are presented in Table 7. These data were compared with drug consumption in Serbia and converted into percentages, enabling the calculation of the percentage difference for each drug within this group.

**Figure 4:**
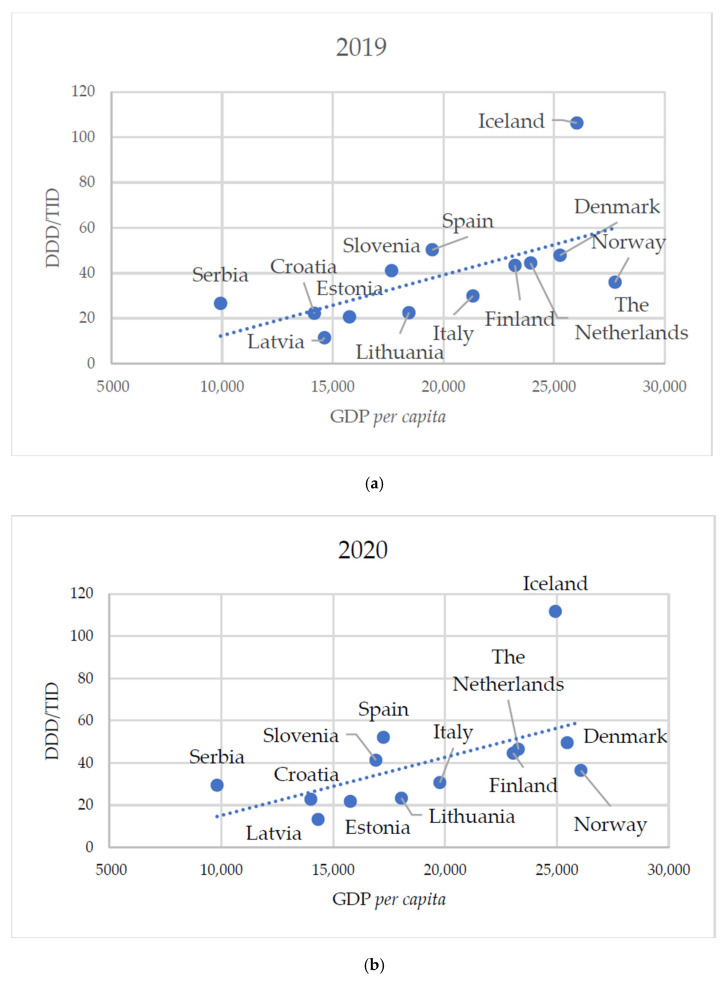
Correlation of annual SSRI group consumption in 13 European countries with their GDP per capita in 2019 (**a**) and 2020 (**b**).

A comparison of total SSRI consumption and GDP per capita across European countries revealed a strong positive correlation in 2019 (ρ = 0.714; *p* = 0.0081), the year with the lowest SSRI consumption, and a moderate positive correlation in 2020 (ρ = 0.659; *p* = 0.0171), the year with the highest consumption (Table 8 and Figure 4).

## 4. Discussion

Since their discovery in the 1990s, SSRIs have become the preferred choice for treating depressive disorders due to their simpler therapeutic application, as well as their milder side effects and lower toxicity compared to tricyclic antidepressants [14,15], which were previously considered first-line treatments. Our analysis showed that sertraline experienced a decrease in consumption in 2019 compared to 2018 (20%), followed by an increase in 2020 (8%) and then a further decrease in 2021 and 2022 (17% and 6%).

Escitalopram experienced constant growth (21%, 14%, 1%, and 14%), making it the most widely used drug by 2022. Paroxetine also showed consistent growth until 2022 (4%, 11%, and 55%), but its consumption decreased by 29% in 2022. Fluoxetine had an increase in consumption in 2019 and 2020 (17% and 21%), followed by a decrease in 2021 (21%), before seeing another increase in 2022 (20%). Citalopram showed a constant decline in consumption (2%, 4%, 4%, and 10%), remaining the least-prescribed drug in this group. Group consumption fluctuated, with a decrease in consumption in 2019 compared to 2018 (7%), followed by an increase in 2020 (11%), and then a decline in 2021 and 2022 (0.3% and 4%). Compared to 2015 [16], there was an increase in the consumption of sertraline (81%), escitalopram (40%), paroxetine (2%), and group SSRI consumption (38%). In contrast, the consumption of fluoxetine (17%) and citalopram (16%) decreased.

The most commonly used SSRI in Serbia from 2018–2021 was sertraline. Sertraline is recommended for patients over 60 years old due to its relatively mild interactions with other drugs, compared to other SSRIs [17]. Furthermore, since the half-life and incidence of side effects of sertraline are similar in pediatric populations and adults, it can be used in children and adolescents following the standard titration regimen for adults [18]. Although both sertraline and its metabolite, DMS, were found in breast milk and infant plasma, their concentrations are low enough to avoid causing adverse effects in infants [19,20]. In patients with renal insufficiency, there are no significant differences in sertraline pharmacokinetics, compared to healthy individuals [21,22]. However, in patients with hepatic insufficiency, clearance is reduced, and the half-life is greatly prolonged, requiring dose adjustments [23]. Significant drug interactions include the potential for sertraline to enhance the anticoagulant effect of warfarin, necessitating the monitoring of INR. Additionally, combining sertraline with lithium may increase the risk of tremors [24]. Increased concentrations of sertraline have also been observed in adolescents who consume marijuana [25].

Escitalopram, which has shown a consistent increase in consumption both in Serbia and globally [26], became the most commonly used SSRI in Serbia in 2022. As the S-enantiomer of citalopram, escitalopram is responsible for all of its therapeutic effects [27]. No statistically significant differences in pharmacokinetics have been found in adolescents, the elderly, or patients with liver insufficiency, compared to the general population [28]. Escitalopram is a weak inhibitor of several CYP isoenzymes, including 1A2, 2C9, 2C19, 2D6, and 3A4, and has a low potential for interactions with drugs metabolized via these enzymes [29]. However, when used in combination with omeprazole, a CYP2C19 and CYP3A4 inhibitor, or cimetidine, a CYP2D6 and CYP3A4 inhibitor, the elimination of escitalopram is reduced [30]. A statistically but not clinically significant prolongation of the half-life of metoprolol has been observed when it is used concurrently with escitalopram [31].

Paroxetine was the third most commonly consumed SSRI in Serbia. Its half-life is prolonged in the elderly and patients with renal insufficiency, so smaller initial doses are recommended for these populations [32]. As a strong inhibitor of CYP2D6, paroxetine can interfere with both its own metabolism [33] and the metabolism of other drugs metabolized by this enzyme, such as metoprolol [34], clozapine [35], desipramine [36], and imipramine [37]. Furthermore, paroxetine may interact with anticoagulant drugs, such as warfarin [38]. Although paroxetine is excreted in breast milk, there is no contraindication to breastfeeding.

Fluoxetine, the first SSRI, was introduced in literature in 1974, making it the oldest drug in this group [39]. It is a potent inhibitor of CYP2D6 [40,41], which can lead to the inhibition of its own metabolism [42], contributing to its long half-life. In patients with hepatic insufficiency, the half-life is further prolonged, necessitating dose adjustments [32]. Since both fluoxetine and its active metabolite, norfluoxetine, are excreted in breast milk, breastfeeding should either be discontinued or conducted with minimal therapeutic doses [43,44]. As a potent CYP2D6 inhibitor, numerous interactions with drugs metabolized via this enzyme are possible, including beta-blockers (atenolol, bisoprolol, and metoprolol), antiarrhythmics (amiodarone), antihypertensives (clonidine), antipsychotics (risperidone and haloperidol), other antidepressants (citalopram, escitalopram, and amitriptyline), and cancer drugs (tamoxifen) [45]. Additionally, fluoxetine can interact with anticoagulant drugs, such as warfarin.

Citalopram was the least frequently used antidepressant among SSRIs in Serbia. It is a racemic mixture composed of two enantiomers, S-(escitalopram) and R-citalopram, in a 1:1 ratio [46]. The S-enantiomer is responsible for citalopram’s effects in inhibiting serotonin reuptake, whereas the R-enantiomer may attenuate this effect through a pharmacodynamic interaction [27,46]. The half-life of citalopram is prolonged in elderly patients and individuals with hepatic insufficiency, warranting dose adjustments in these populations [32].

Among the 12 European countries in 2019 and 2020, 8 had higher consumption than Serbia (Italy, Iceland, Spain, Norway, Netherlands, Finland, Slovenia, and Denmark), and 4 had lower consumption (Croatia, Estonia, Latvia, and Lithuania). Based on the percentage difference in SSRI consumption relative to Serbia, countries with higher usage were categorized into four groups: Group 1 (0–25%), Group 2 (25.1–50%), Group 3 (50.1–75%), and Group 4 (>75%). In Group 1, Italy remained in both years with percentage differences of 13% and 4%. Norway, initially in Group 2 in 2019 with a 35% higher consumption, shifted to Group 1 in 2020 (24%). The Netherlands and Finland consistently belonged to Group 3, with respective differences of 67% and 58% for the Netherlands and 63% and 52% for Finland in 2019 and 2020. Slovenia, which was also in Group 3 in 2019 (54%), transitioned to Group 2 in 2020 (40%). Spain remained stable in Group 4 (89% and 77%), while Denmark showed a decrease, moving from Group 4 to Group 3 (80% and 68%). Iceland, the country with the highest global consumption of SSRIs, reported levels approximately 300% higher than Serbia in 2019 and 280% higher in 2020. Among the countries with lower SSRI consumption than Serbia, two groups were defined: Group 1 (0–25%) and Group 2 (>25.1%). Croatia (16% and 23%) and Lithuania (15% and 21%) remained within Group 1 in both years, indicating relatively stable trends. Estonia transitioned from Group 1 (23%) to Group 2 (26%), reflecting a relative decrease. Latvia, which had the lowest SSRI consumption among the 13 selected countries, reported levels that were 57% and 55% lower.

From the twelve European countries analyzed, three were selected for detailed comparison based on their SSRI consumption relative to Serbia: two with higher consumption (Denmark and Spain) and one with lower consumption (Latvia). In Denmark, sertraline consumption was 135% higher in 2019 and 142% higher in 2020, while escitalopram use was 41% and 48% lower, respectively. Citalopram consumption in Denmark was significantly higher, with differences of 1363% in 2019 and 1329% in 2020. In Spain, sertraline usage exceeded Serbia’s by 32% and 33%, escitalopram by 81% and 64%, and citalopram by 543% and 569%, respectively. In contrast, Latvia demonstrated significantly lower consumption: sertraline use was 87% and 86% lower, escitalopram 28% and 26% lower, while citalopram was 9.5% lower in 2019 but 3% higher in 2020, compared to Serbia.

The utilization of SSRIs appears to be associated with the economic status of a country. Findings from our study indicate that countries with a higher gross domestic product (GDP) tend to exhibit greater SSRI consumption, whereas existing literature suggests a decline in SSRI use during times of economic downturn [47]. These findings suggest that countries with greater economic power may demonstrate a stronger recognition of the importance of mental health care and depression treatment. However, even in such settings, periods of economic crisis appear to deprioritize mental health, reflecting the vulnerability of psychiatric care to broader economic pressures. One of the potential reasons for differences in antidepressant consumption between countries may be the degree of stigma surrounding mental disorders in the general population. One-third of respondents in Serbia reported that they would not be willing to live with a person experiencing mental health problems [48]. In addition to stigma, another major barrier to seeking mental health support is physical access—primarily the distance to mental health professionals, long waiting lists, and the high cost of services [48].

## 5. Conclusions

The overall consumption of antidepressants from the SSRI group in Serbia fluctuated between 2018 and 2022, without reaching statistical significance. However, significant trends were observed in the consumption patterns of individual SSRIs, particularly sertraline, escitalopram, and citalopram. Sertraline was the most frequently prescribed SSRI from 2018 to 2021, after which escitalopram use showed a steady increase, becoming the most consumed SSRI in 2022, both in Serbia and globally. When compared to twelve other European countries, Serbia ranked in the mid-range, with eight countries demonstrating higher SSRI consumption and four showing lower levels. Notably, Iceland had the highest SSRI consumption, while Latvia had the lowest among the selected countries. Additionally, a positive correlation was found between SSRI use and GDP per capita across 13 European countries, suggesting that economic factors may influence antidepressant consumption. Further research into regional prescribing patterns, socioeconomic factors, and clinical guidelines could help improve mental health treatment and ensure equal access to effective antidepressant therapy.

## Figures and Tables

**Table 1:** Therapeutic indications for SSRI medications.

SSRI	Depression	Panic Attacks	OCD	Social Phobia	GAD	PTSD	Bulimia Nervosa	PTSD
*fluoxetine*	X (8+)	X	X (7+)	X		X	X	X
*citalopram*	X							
*paroxetine*	X	X	X	X	X	X		X
*sertraline*	X	X	X (6+)	X		X		X
*escitalopram*	X (12+)				X			

OCD—obsessive–compulsive disorder, GAD—generalized anxiety disorder, PTSD—post-traumatic stress disorder, PMDD—premenstrual dysphoric disorder.

**Table 2:** Pharmacokinetics of SSRIs.

SSRI	Time to Maximum Concentration	Bioavailability	Bound Fraction	Volume of Distribution	Half-Life
*fluoxetine*	6–8 h	60–80%	94%	20–40 L/kg	96 h
*citalopram*	2–4 h	80%	80%	12–16 L/kg	30–35 h
*paroxetine*	5–6 h	30–60%	95%	3–12 L/kg	21 h
*sertraline*	4–8 h	44%	98%	25 L/kg	22–36 h
*escitalopram*	3–4 h	80%	56%	12–26 L/kg	27–33 h

**Table 3:** Consumption of antidepressants from the SSRI group in the period 2018–2022, expressed in defined daily dose per 1000 inhabitants per day (DID).

Medicine	DID 2018	DID 2019	DID 2020	DID 2021	DID 2022
*fluoxetine*	2.40943	2.81899	3.40094	2.68469	3.22032
*citalopram*	0.97396	0.95170	0.91167	0.87061	0.78152
*paroxetine*	4.16225	4.32504	4.79192	7.40698	5.25851
*sertraline*	14.8493	11.0207	11.8468	9.85769	9.24536
*escitalopram*	6.13398	7.44041	8.46109	8.51289	9.73523

**Table 4:** The trend of antidepressant consumption from the SSRI group in the period 2018–2022 in the Republic of Serbia.

Medicine	Trend Equation	R^2^	Adjusted R^2^	*p*-Value
*fluoxetine*	y = 0.1487x + 2.4606	0.342	0.1227	0.300
*citalopram*	y = −0.0466x + 1.0377	0.9376	0.9168	0.007 *
*paroxetine*	y = 0.5274x + 3.6066	0.4042	0.2056	0.249
*sertraline*	y = −1.2371x + 15.075	0.7948	0.7263	0.042 *
*escitalopram*	y = 0.8275x + 5.5742	0.9424	0.9233	0.006 *
**SSRI**	y = 0.22x + 27.754	0.0908	−0.2123	0.622

R^2^—Coefficient of determination; *—statistically significant values (*p* < 0.05).

**Table 5:** Annual group SSRI consumption in the Republic of Serbia, expressed in defined daily dose per 1000 inhabitants per day (DID).

Year	DID SSRI
2018	28.52887
2019	26.55683
2020	29.41245
2021	29.33288
2022	28.24096

**Table 6:** Annual group consumption of SSRIs in the Republic of Serbia and 12 European countries, expressed in defined daily dose per 1000 inhabitants per day (DID).

Country	DID 2018	DID 2019	DID 2020	DID 2021	DID 2022
Serbia	28.53	26.56	29.41	29.33	28.24
Italy	29.70	29.90	30.6	31.20	31.70
Iceland	103.3	106.3	111.7	118.9	116.3
Spain	49.09	50.27	52.11	55.07	57.71
Croatia	21.66	22.19	22.69	23.85	24.61
Norway	35.46	35.97	36.35	37.58	/
The Netherlands	42.35	44.41	46.5	48.21	49.71
Estonia	18.91	20.58	21.72	24.06	26.26
Finland	41.39	43.40	44.58	46.45	/
Latvia	10.42	11.45	13.17	14.26	15.76
Lithuania	20.127	22.50	23.22	23.86	25.74
Slovenia	40.30	41.00	41.30	42.90	44.20
Denmark	46.70	47.80	49.50	52.50	55.20

**Table 7:** Defined daily dose per 1000 inhabitants per day (DID) for individual SSRI medications in Serbia, Denmark, Spain, and Latvia in 2019 and 2020 (based on available data).

Title 1	Serbia		Denmark		Spain		Latvia	
	2019	2020	2019	2020	2019	2020	2019	2020
*fluoxetine*	2.82	3.4	2	1.8	6.94	7.06	0.51	0.63
*citalopram*	0.95	0.91	13.9	13	6.11	6.09	0.86	0.94
*paroxetine*	4.33	4.79	2.2	2.1	8.8	8.9	3.1	3.52
*sertraline*	11.02	11.85	25.9	28.7	14.56	15.78	1.4	1.63
*escitalopram*	7.44	8.46	4.4	4.4	13.46	13.88	5.39	6.24

**Table 8:** Annual group consumption in 13 European countries, expressed in defined daily dose per 1000 inhabitants per day (DID), and their GDP per capita in 2019 and 2020.

Country	DID SSRI (2019)	GDP Per Capita (2019)	DID SSRI (2020)	GDP Per Capita (2020)
Serbia	26.56	9927.1	29.41	9809.7
Italy	29.90	21,336.7	30.60	19,772.8
Iceland	106.3	26,029.8	111.7	24,937.5
Spain	50.27	19,497.2	52.11	17,253.5
Croatia	22.19	14,168.2	22.69	14,004.4
Norway	35.97	27,761.2	36.35	26,073.7
The Netherlands	44.41	23,939.2	46.50	23,281.6
Estonia	20.58	15,760.0	21.72	15,771.9
Finland	43.40	23,237.4	44.58	23,047.9
Latvia	11.45	14,628.4	13.17	14,325.5
Lithuania	22.50	18,444.7	23.22	18,053.4
Slovenia	41	17,652.1	41.30	16,907.5
Denmark	47.80	25,277.4	49.50	25,457.2

## Data Availability

The data presented in this study are available on request from the corresponding author.

## Abbreviations

The following abbreviations were used in this manuscript:

DDD	Defined daily doses
DDD/TID	Defined daily doses per 1000 inhabitants per day
